# Impact of standalone and embedded telephone triage systems on after hours primary medical care service utilisation and mix in Australia

**DOI:** 10.1186/1743-8462-2-30

**Published:** 2005-12-12

**Authors:** David Dunt, Susan E Day, Margaret Kelaher, Michael Montalto

**Affiliations:** 1 Program Evaluation Unit, School of Population Health, University of Melbourne, 3010, Australia; 2 Epworth Hospital, Bridge Rd, Richmond, Vic, 3121, Australia

## Abstract

**Background:**

The Australian government sponsored five local trials aimed at addressing problems in after hours (AH) primary medical care (PMC). The study's objective was to determine if the four trials, where telephone triage was the sole innovation, led to a reduction in AH service utilisation and change in service mix towards AH GP clinics. Changes in utilisation and mix of AH GP clinic and home visits, ED and ambulance use were monitored in the trial areas, and in a national sample to adjust for the effects of secular trend. Pre- and post-trial telephone surveys of two separate random samples of approximately 350 AH PMC user households in each area were conducted.

**Results:**

Some types of AH PMC use became more frequent in both of the standalone services using nurse-administered proprietary call centre software, which were aimed at better addressing population need (*Statewide call centre; Regional call centre*). Service use overall (95%CI: 1.03–1.83) and GP clinic use (95%CI: 1.07–2.00) increased in the metro area of the *Statewide call centre *and in GP clinic (95%CI: 1.04–2.14) and home visits (95%CI: 1.03–3.91) in the non-metro area of the *Regional call centre*. Service mix only changed in the non-metro area of the *Regional call centre *with increased contact in GP home visits (95%CI: 1.02–4.38). Levels of use remained unchanged in both embedded services using other than proprietary software, which were established to support the GP workforce (*Deputising service; Local triage centre*). Service mix only changed in the *Deputising service *with a change away from AH GP clinics in both contact (95%CI: 0.39–0.97) and frequency (95% CI: -2.12 – -0.7).

**Conclusion:**

Bearing in mind limitations in estimating AH PMC utilisation levels and mix, it is concluded that the impacts of telephone triage were generally smaller in Australia than reported elsewhere. There were different impacts on levels of service utilisation and service mix in standalone call centres and embedded services. Impacts of telephone triage on service utilisation and mix are influenced by the type of telephone triage offered, the goals of the agency providing the service, as well as local factors. (345 words)

## Background

Models of AH PMC are in flux in a number of developed countries. In Australia there is increasing concern that lack of AH care is resulting in an increase in care substitution with more GP-like presentations occurring in emergency departments [1]. While it remains unclear whether this is true, it has resulted in a renewed impetus to develop viable models for providing high quality AH PMC.

Telephone triage and advice services form one common model or feature of care in the provision of AH PMC. Most frequently these services are embedded in other AH services such as general practitioner (GP) cooperatives in the UK, HMOs in the US and the county-based service arrangements now operating in Denmark [2]. In addition, a small number of standalone services have been established. NHS Direct, the national AH telephone triage service system in the UK, based on nurses using proprietary health call centre software is the most important of these. An outcome of the recent review of AH services in the UK is that NHS Direct has become the point of first contact for people accessing AH services in the UK. It is now also better integrated with other AH service providers, and not just GPs, and operates with improved functionality [3].

A recent structured review of AH PMC care was however only able to identify a small number of high quality studies evaluating the various impacts of these telephone triage services [4]. Central to these impacts is their effect on service utilisation, given that high levels of service utilisation pose the greatest challenge to the viability of AH PMC services. The review concluded that the growth in telephone triage and adviceservices usually, but not always, reduced immediatemedical workload through the substitution of telephone callsfor in-person consultations. Considering embedded services first, a randomised control trial (RCT) from the UK compared a nursetelephone consultation service integrated within a GP co-operative with the usual practiceof that co-operative [5]. There was 69% reduction in telephone advicefroma GP, 38% reduction in patient attendanceatprimary care centres and 23% reduction in home visits.

The implementationof a telephone-based nurse triage service in a HMO, in the US (where GPs do not act as gatekeepers to medical care) led to 15% decrease in hospitalemergency department services and 11% decrease in out-patient physicianservices [6]. AH telephoneaccess to physicians in an inner city, adult, general medicine clinic again in the US, had no impact on hospitalisations or ED visits, as demonstrated by an RCT. Uptakeof the service thoughwas low [7].

In 1992 in Denmark, locally organised GP AH serviceswere replaced with centrally organised services that includedtelephone triage and advice services. Christensen found that the numberof consultations in doctors' surgeries was relativelyunchanged, but home visits were much reduced from46 to 18% [8]. Hansen, in the county of Funen, three yearsafter the change reported similar results [9].

Considering standalone services, a before/after study following the introduction of NHS Direct in the UK found a small, but significant change in use of GP co-operatives but no change in use of ED and ambulance services [11]. NHS Direct also had no impact on the number of general practice consultations for influenza-like illness and other respiratory infections during a winter epidemic[12]. A pilot national telephone advice service (Healthline) in Christchurch, New Zealand though had little effect on overall ED numbers but decreased the workload for ED nursing staff charged with answering advice calls [10].

This article considers a recent initiative of the Australian Government – the After Hours Primary Medical Care Trials (AHPMCTs) and its impact on service utilisation and service mix. The goals of the AHPMCTs were to improve the quality of service delivery as well as consumer acceptability, consumer access (including affordability) and equity, appropriateness of service mix, provider satisfaction with regard to their impact on service mix as well as service use more generally [13].

The aim of this paper is to determine in four of the five AHPMCTs where telephone triage was the only service innovation, whether telephone triage reduced immediate AH PMC service utilisation and altered AH service mix towards GP clinic use (and away from GP home visits, ED visits and ambulance use). Two of these four AHPMCTs were standalone services and the two others, embedded services. It is therefore possible to compare the effects of telephone triage in standalone and embedded services on service utilisation and service mix. The study covers not just GP care – GP clinic care and GP home visits – but also ED, ambulance and professional medical advice by telephone. Service utilisation analysis is considered at a whole population level – rather than at an agency level – which surprisingly, is uncommon in previous studies of AH PMC care [8,14]. In these ways the article adds to the growing knowledge base on the effects of telephone triage on AH PMC services.

### The AHPMCTs

Telephone triage services used by the four study AHPMCTs differed in form and function within the individual AHPMCT as follows.

### Standalone services

These were call centres where nurses, using proprietary health call centre software, which were aimed at providing more accessible advice and promoting more appropriate AH PMC service use.

1. A stand-alone *Statewide call centre *(studied in both metro and non-metro areas);

2. A stand-alone *Regional call centre *(studied in both inner metropolitan and rural satellite areas);

### Embedded services

These triage and advice systems did not use proprietary health call centre software. The aims of the services in which they were embedded were also different. These were to manage demand so as to support the GP workforce in terms of recruitment and retention. They consisted of

3 A GP-based telephone triage and advice service (without guidelines or software) in a pre-existing *Deputising service*;

4 *Local triage *and advice service using hospital nurses with locally developed, paper-based protocols to support AH services in GP own-practice arrangements in a small rural community;

The AHPMCTs commenced operation in late 1999-mid 2000. Their organisational settings and regional contexts are summarised in Table 1.

**Table 1:** Local context and services of individual trials

**Trial**	Catchment Areas * †	**Notes**
1. Statewide call centre	Capital city, small rural centre and Other rural area 1.42 M Rest of state – small rural centre and other rural area studied 42,500	1 Operator with prior international industry experience under contract to the State Government) 2 Free to caller
2. Regional call centre	Capital city (inner region) 537,000 Non-metro satellite: Small rural centre 21,000	1 Startup operator 2 Free to caller 3 A GP AH walk-in clinic located adjacent to the ED of a regional hospital in the central metro area operating for part of the study period closed due to lack of demand.
3. Deputising service	Capital city and Other rural area 229,000	1 GP-based telephone triage and advice service 2 Pre-existing and continuing *Deputising service *offering an AH walkin clinic and home visiting service. 3 Service offered to patients of previously enrolled GP practices and new enrolled GP practices beyond the metro area. 4 Commonwealth government funding of the program derived from 'cashing out' historical MBS reimbursements to the service – home visits during the trial not billed to patient.
4. Local triage and advice service	Other rural area (ie small country town and surrounding community) 21,000	1 Hospital nurses using locally developed paper-based protocols 2 located in a local hospital in a rural community. 3 Aim to support GP own-practice AH arrangement to relieve GP AH burden.

* Based on Rural, Remote and Metropolitan Areas classification (RRMA) [17]

**† **Source: Australian Bureau of Statistics population projections for 1999 supplied to the Department of Health and Ageing

## Methodology

### Study design

The national evaluation consisted of 'multiple trials' with common questions and hypotheses, rather than a 'multicentred' trial with common protocols [10]. This reflected the underlying goals of the AHPMCTs which were to meet local circumstances and needs and to investigate different models and types of AH PMC care that were appropriate to these local circumstances. The individual trials were therefore studied separately. While they are not directly compared, their relative success is considered.

A monitoring strategy was adopted, using a pre-post design to detect changes in relevant variables in the trial area across the 12-month study period. To examine whether these changes could reflect period effects occurring nationally, changes occurring in the trial area were compared with those occurring in the rest of Australia outside of AHPMCTs areas (the *National Comparator*).

### Data collection

The regional impacts were measured using telephone surveys of separate random samples of AH user households in trial areas and the national comparator at the beginning of the study period (pre-trial Nov–Dec 1999) and repeated 12 months later (post-trial). The surveys were conducted by a commercial market survey firm under the direction of the evaluation team. Random samples of households were drawn from the latest version of the electronic white pages with each selected household receiving a letter explaining the survey prior to contact by telephone. Once contact had been established with a selected household, interviewers asked to speak to someone 'aged 18 years or over who would know about the use of medical services' in the household (the informant).

If the informant was agreeable, the interviewer then established whether someone in the *household *(not individual) had used an AH medical services during the past year. This information was used to estimate the overall utilisation of AH PMC services in that population. Informants from all user households were then interviewed concerning immediate effects on all AH PMC services, including the frequency of use of each type of service. The volume of calls to the telephone triage system in individual sites was recorded to better understand to what extent changes in utilisation rates were affected by take-up of the new services offered.

### Analysis

Sample size for user households was estimated at 350 per site based on an hypothesised 15% change in service mix to GP clinic from GP home visits, ED and ambulance (80% power, 0.05 level of significance). AH participation rates as a percentage of contacted households at pre- and post-trial are shown in Table 2.

**Table 2:** Number of interviews and response rates for AH user households*

**REGION**		**PRE-TRIAL**		**POST-TRIAL**	
		**Interviews**	**Response Rate**	**Interviews**	**Response Rate**
Statewide Call	Metro	351	73%	350	68%
Centre	Non-Metro	350	71%	350	83%
Regional Call	Central-Metro	349	51%	351	63%
Centre	Satellite	350	76%	350	80%
Deputising service		380	83%	356	76%
Local triage center		166^#^	90%	175^#^	84%
National Comparator		366	77%	351	71%

Notes: * As a percentage of households able to be contacted by telephone

^# ^Reduced numbers due to an error in data collection at baseline. This will have the effect of reducing power to detect 15% changes in use levels in the Local triage centre.

Questions in the interview schedule covered a number of topics relating to AH PMC care including AH use and frequency of AH use by the household in the previous 12 months for GP clinic care, GP home visits, EDs, and ambulance. Questions relating to household's and informant's characteristics were also included.

The effects of the AHPMCTs were analysed in relation to changes across the study period in:

#### i) Characteristics of the households and informants within the user households

Changes in these characteristics alone could affect service outcomes (bearing in mind the possibility of sampling error). Six characteristics of the user and four of their household at pre-trial and post-trial in each trial area (metro and non-metro, separately where appropriate) and national comparator were compared to test for this – see Table 3.

**Table 3:** Characteristics of the study population

	**STATEWIDE CALL CENTRE**		**REGIONAL CALL CENTRE**		**DEPUTISING SERVICE**	**LOCAL TRIAGE CENTRE**	**NATIONAL COMP**
	**Metro**	**Non-metro**	**Metro**	**Non-Metro**			
**INFORMANTS**							
**Average Age (95% CI)**							
Baseline	46 (44.2–47.5)	45 (43.2–476.5)	44 (42.2–45.8)	49 (47.5–51.1)	47 (45.4–58.6)	49 (46.1–50.1)	46 (43.8–47.0)
Follow-up	47 (45.3–48.6)	47 (44.9–48.2)	46 (44.3–47.6)	49 (47.7–51.2)	48 (46.6–50.0)	50 (47.3–52.1)	46 (44.0–47.2)
**Gender (% Female)**							
Baseline	67	71	60	66	66	68	67
Follow-up	70	70	64	70	71	65	68
**Overseas Born (%)**							
Baseline	27	18	35	5	12	6	9
Follow-up	37*	9	40	9	13	4	7
**Indigenous Origin (%)**							
Baseline	1	2	4	3	2	0	3
Follow-up	1	1	1*	1	2	0	1
**Health less than 'good' (%)**							
Baseline	20	25	14	36	26	24	21
Follow-up	17	25	14	25	27	23	21
**Financially Disadvantaged (%)**							
Baseline	20	15	19	24	28	42	18
Follow-up	22	19	21	19	24	35	19
							
**HOUSEHOLDS**							
**Average Number of Persons in the Household (95% CI)**							
Baseline	3.4 (3.2–3.5)	3.5 (3.3–3.6)	3.0 (2.9–3.2)	2.8 (2.7–3.0)	3.1 (2.9–3.2)	2.8 (2.6–3.1)	3.3 (3.2–3.5)
Follow-up	3.3 (3.2–3.5)	3.3 (3.1–3.4)	3.1 (2.9–3.2)	2.9 (2.7–3.0)	3.1 (2.9–3.2)	3.0 (2.8–3.3)	3.3 (3.1–3.4)
**Single Person Household (%)**							
Baseline	9	10	17	21	16	21	12
Follow-up	10	15	16	15	11	14	11
**Children Under 12 Years of Age in the Household (%)**							
Baseline	43	47	33	36	37	34	36
Follow-up	38	41	36	33	33	36	42
**Someone in the Household with a Chronic Illness or Disability (%)**							
Baseline	25	25	19	31	29	25	21
Follow-up	21	22	21	31	30	31	22

* P < 0.05

#### ii) Service utilisation

Changes in overall levels of contact with AH PMC services in the *whole *population (ie users and non-users) in each site were compared – see Table 4. Changes in each site were also compared with the national comparator using multiple logistic regression analysis to control for the effects of secular trend – see Table 5. The principal independent variables considered were: time (pre/post trial), intervention status (trial area/national comparator), and trial effect (time*intervention status). It was not possible to incorporate informant and household characteristics in this analysis as data on these was only available for a quota of 110 non-users, rather than all the non-user households/informants in each area. This being the case, analysis consisted of interpreting changes in use in a trial, area, corrected for secular trend, in the light of changes in the characteristics of the baseline and follow-up population, as described immediately above.

**Table 4:** Contact with AH services in whole population in each AHPMCT area and national comparator

	**STATEWIDE CALL CENTRE**		**REGIONAL CALL CENTRE**		**DEPUTISING SERVICE**	**LOCAL TRIAGE CENTRE**	**NATIONAL COMPARATOR**
	**Metro**	**Non-metro**	**Metro**	**Non-metro**			
Households contacted							
Baseline	776	801	739	741	824	378	738
Follow-up	724	897	806	753	838	531	784
Households using AH services (overall utilization rate)							
Baseline	45%	44%	47%	47%	46%	44%	50%
Follow-up	48%	39%^#^	44%	46%	42%	34%***	45%^#^
Household using a GP clinic AH							
Baseline	25%	19%	33%	13%	32%	25%	33%
Follow-up	30%	20%	28%*	16%^#^	25%***	21%	30%
Households having a GP home visit							
Baseline	5%	2%	10%	4%	9%	4%	6%
Follow-up	5%	2%	6%**	5%	6%*	2%^#^	4%*
Households visiting the ED							
Baseline	31%	36%	22%	49%	28%	31%	30%
Follow-up	30%	32%	23%	42%	27%	24%*	28%^#^
Households using ambulance services AH							
Baseline	6%	4%	6%	10%	8%	5%	7%
Follow-up	6%	5%	7%	9%	8%	4%	6%

* P < 0.05; ** P < 0.01; *** P < 0.001; ^# ^0.05 < P < 0.10

**Table 5:** Change in contact in the whole population in AHPMCT area compared with the national comparator (adjusted odds ratio & 95% Confidence Intervals [CIs]

	STATEWIDE CALL CENTRE		REGIONAL CALL CENTRE		DEPUTISING SERVICE	LOCAL TRIAGE CENTRE
	Metro	Non-metro	Metro	Non-metro		
*Overall use*	1.38 (1.03 – 1.83)	1.00 (0.76 – 1.32)	1.04 (0.79 – 1.39)	1.18 (0.88 – 1.57)	1.05 (0.79 – 1.39)	0.76 (0.54 – 1.07)
*All GP*	1.56 (1.15 – 2.12)	1.29 (0.94 – 1.77)	0.91 (0.68 – 1.23)	1.51 (1.08 – 2.12)	0.92 (0.68 – 1.24)	0.98 (0.68 – 1.42)
*GP clinic*	1.46 (1.07 – 2.00)	1.18 (0.86 – 1.64)	0.94 (0.69 – 1.28)	1.49 (1.04 – 2.14)	0.82 (0.61 – 1.11)	0.94 (0.65 – 1.38)
*GP home visit*	1.75 (0.90 – 3.39)	1.74 (0.79 – 3.85)	0.98 (0.53 – 1.79)	2.01 (1.03 – 3.91)	1.10 (0.61 – 1.99)	0.96 (0.34 – 2.15)
*ED*	1.08 (0.79 – 1.47)	0.92 (0.69 – 1.25)	1.19 (0.86 – 1.65)	1.12 (0.82 – 1.51)	1.10 (0.80 – 1.49)	0.77 (0.53 – 1.11)
*Ambulance*	1.07 (0.60 – 1.92)	1.26 (0.68 – 2.32)	1.31 (0.74 – 2.33)	1.01 (0.59 – 1.71)	1.19 (0.70 – 2.04)	0.98 (0.46 – 2.10)

#### iii) Service mix

Any contact with AH PMC services (GP clinic, GP home visit, ED) in the *user *population was assessed using multiple logistic regression analysis – see Table 6. OLS regression was used for studying the frequency of this contact. The ten informant and household characteristics potentially having effects on service utilisation, were also included in the analysis.

**Table 6:** Change in service mix in the user population in AHPMCT area compared with national comparator(change in contact: adjusted odds ratio & 95% Confidence Intervals [CIs]; frequency of contacts – unstandardised B coefficients [beta standardised in square brackets] & 95% CIs for unstandardised coefficients)

	**STATEWIDE CALL CENTRE**		**REGIONAL CALL CENTRE**		**Deputising service**	**Local triage centre**
	**Metro**	**Non-metro**	**Metro**	**Non-metro**		
*GP clinic visit*						
Contact	1.35 (0.86 – 2.12)	1.38 (0.89 – 2.14)	0.94 (0.59 – 1.50)	1.42 (0.90 – 2.24)	0.62* (0.39 – 0.97)	1.49 (0.85 – 2.62)
Frequency	0.07 [0.01] (-1.14 – 1.28)	-0.32 [-0.03] (-1.40 – 0.77)	-0.84 [-0.07] (-2.03 – 0.36)	0.33 [0.03] (-0.90 – 1.56)	-1.09 [-0.11] (-2.12 – -0.7)^†^	1.20 [0.09] (-0.25 – 2.66)
*GP home visit*						
Contact	1.37 (0.67 – 2.80)	1.70 (0.73 – 3.94)	0.78 (0.40 – 1.51)	2.11^‡ ^ (1.02–4.38)	1.06 (0.55 – 2.01)	0.96 (0.36 – 2.58)
Frequency	0.60 [0.17] (-0.20 – 1.41)	0.47 [0.12] (-0.44 – 1.37)	0.36 [0.09] (-0.61 – 1.32)	0.66 [0.15] (-0.41 – 1.71)	0.23 [0.05] (-0.90 – 1.36)	0.42 [0.10] (-0.49 – 1.32)
*ED visit*						
Contact	0.78 (0.50 – 1.22)	0.99 (0.60 – 1.64)	1.19 (0.77 – 1.84)	1.11 (0.63 – 1.98)	1.17 (0.76 – 1.82)	0.99 (0.56 – 1.74)
Frequency	-0.31 [-0.05] (-1.03 – 0.41)	0.12 [0.002] (-0.77 – 0.81)	0.08 [0.01] (-0.67 – 0.83)	-0.15 [-0.02] (-1.03 – 0.72)	0.02 [0.003] (-0.71 – 0.76)	0.30 [0.03] (-0.80 – 1.34)

**Notes: **Other variables having independent effects where the effect of the AHMCT is statistically significant

* age (0.97–0.98); Persons over 12 years in household (0.49–0.99) [Odds ratio]

^‡ ^age (1.02–1.05); Informant Overseas-born (1.24–3.24); Persons in household (1.09–2.73); Person with chronic illness in household (1.07–2.36) [Odds ratio]

^† ^Indigenous origins (0.14–3.89); Informant Overseas-born (0.24–1.68), Children under 12 years in household (0.15–1.51); Person with chronic illness in household (0.85–2.05) [B coefficient]

No adjustment was made for multiple comparisons as the study was more hypothesis-generating than testing in character. SPSS for Windows version 12.0.1 was used. In interpreting results, it should be noted that the service in the metro area of the *Statewide call centre *had been operating for around five months before the pre-trial survey was conducted and important effects on utilisation may not have been detected. A GP AH walk-in clinic, adjacent to the ED of a regional hospital operated by the *Regional call centre *during part of the trial period in its central metro area but closed due to lack of demand.

## Results

### National comparator

There was no change in *National comparator *household or informant characteristics across the trial period. GP home visits alone decreased (alongside a non-significant reduction in overall AH PMC service use) in the *whole population *of users and non-users.

### Standalone services

#### 1 *Statewide call centre *(metro and non-metro) – see Tables 4, 5 and 6

Only one of ten informant or household characteristics changed post-trial in the metro study area (there was a higher proportion of overseas-born informants) and none in the non-metro area. Approximately 50 AH PMC calls/month/10,000 head of population were made in the metro area over the first nine months of operation of the service. No call centre monitoring data was available for the non-metro area [10].

There was no reduction in overall or particular type of AH PMC utilisation pre/post in either the metro or non-metro areas of the *Statewide call centre *in the whole population of users and non-users. There was though, when compared with the *National comparator*, a change in overall AH service use and in All GP use due to an increase in GP clinic use in the metro area. Since only one informant and no household characteristic changed in the metro area of the *Statewide call centre *and none in the *National comparator*, it is very likely that this is a direct trial effect. Service mix (any contact as well as frequency of contact among the user population) did not change compared with the *National comparator*.

#### 2 *Regional call centre *(inner metropolitan and rural satellite) – see Tables 4, 5 and 6

One informant characteristic changed post-trial in the metro area (there was a smaller proportion of Indigenous informants). There were 18 AH PMC calls/month/10,000 head of population in the metro area and six in the satellite area during the first 12 months of operation [10]. GP clinic visits and home visits in the metro area alone decreased in the whole population of users and non-users pre-/post. However, when compared with the National comparator, these changes were not significant. There was no change in GP clinic visits and home visits in the metro area pre-/post. However, when compared with the *National comparator, GP *clinic and home visits increased in the non-metro area (also leading to a significant increase in All GP). There was no change in informant or household characteristics in the non-metro area or national comparator across the study period, so this increase can also be regarded as a direct trial effect.

Service mix (any contact as well as frequency of contact among the user population) did not change compared with the *National comparator *except for an increase in contacts for GP home visits in the non-metro area.

### Embedded services

#### GP-based telephone triage and advice service in pre-existing *Deputising service *– see Tables 4, 5 and 6

There were no changes in informant or household characteristics across the trial period. Approximately 31 AH PMC calls/month/10,000 head of population were made [10]. There was a statistically significant decrease in use of AH GP clinic and GP home visits in the whole population of users and non-users at this site pre-/post. When compared with change in the *National comparator *though, this change was not statistically significant. Service mix changed with a reduction in both any contact and frequency of contact for GP clinic in the user population compared with the *National Comparator*.

#### 3 *Local triage *and advice service using hospital nurses with locally developed paper-based protocols – see Tables 4, 5 and 6

There were no changes in informant or household characteristics across the trial period. Approximately 24 AH PMC calls/month/10,000 head of population were made [10]. There was a substantial, statistically significant reduction in overall AH PMC contact associated with reduced ED visits in the *whole *population of users and non-users at this site. This change was not statistically significant however when compared the *National comparator*. Service mix did not change for any contact or frequency of contact for any AH PMC service type in the user population when compared with the *National comparator*.

### Other features of AH PMC service utilisation

None of the AHPMCTs had any impact on level of reported use of ambulance services or telephone professional medical advice.

GP clinic and ED were the most commonly used AH services across the AHPMCTs and were typically used by one quarter to one third of all households (approaching one half in the non-metro area of the *Regional call centre*). GP home visits and ambulance use occurred much less commonly. GP clinics were used somewhat more commonly than ED in metropolitan areas, and not surprisingly the opposite was true in non-metropolitan areas. Between 15% and one third of all households received professional telephone advice.

## Discussion

The following limitations of the study should be noted. The trials were monitored, and took into account secular trend only at the national level. Assessment of impacts were based on self-reported rather than objective data on service utilisation and illness. The study may also have failed to detect changes in the *Local triage centre *– see Table 2. To complement these findings, we will present objective data in follow-up publications based on changes across the trial period in MBS AH fees in AHPMCT sites and nationally as well as ED and ambulance data. These too though have several limitations. MBS items in 2000–1 covered only emergency (GP first call-out) not routine AH care. Reporting of ED and ambulance care events across the country was also very variable both in quality and the nature of data recorded making comparisons of only limited use. Changes in MBS usage for emergency AH care though, was very similar to results presented here.

Standalone and embedded AHPMCTs had different impacts overall on levels of service utilisation. There was evidence of increased use in both standalone call centres with neither showing change in service mix. By contrast, both embedded services showed no significant changes in service use. In addition, the *Deputising service *showed a change in service mix away from AH GP clinics. Thus, a central trial objective that there would be a change in service mix towards the use of GP AH clinic use was not confirmed in any AHPMCT.

The magnitude of effects for standalone services are similar to those for NHS Direct but are in the opposite direction (increase rather than decrease). The magnitude of effects for embedded services is smaller than in the results for most, but not all such studies in the structured review [4]. This in part reflects the nature of the sample population (drawn from the whole population of a defined area rather than users of a particular service alone). The magnitude of effects will also be affected by the take-up of the services – the number of callers using the telephone services. The telephone triage call rate in the four AHPMCTs varied – from 50 AH PMC calls/month/10,000 population in the metro area of the *Statewide call centre *to six in the non-metro area of the *Regional call centre*, possibly relating to the level of marketing and promotion. The uptake of telephone triage, with the exception of the *Regional call centre *(non-metro area),*prima facie *should have been sufficient to produce an effect on utilisation levels and service mix. In fact effects were observed for the non-metro area of the *Regional call centre *which may have been greater if take-up had been greater.

These Australian findings, presented for the first time, reinforce the conditionality of the structured review's conclusion that the growth in telephone triage and adviceservices usually, but not always, reduces immediatemedical workload through the substitution of telephone consultationsfor in-person consultations [4].

The findings are consistent with the studies in the structured review that demonstrate greater reduction in levels of utilisation for embedded than standalone services [4]. One possible reason for this is provided by a cluster RCT which demonstrated that telephone triage, when provided in the patient's own general practice, had a higher resolution rate that was less likely to lead to a GP appointmentthan when provided by NHS Direct [15]. A second possible reason is that standalone and embedded services were established with somewhat different aims. The embedded services were concerned with managing demands in relation to industry needs whereas the standalone services were aimed at establishing a new type of service to better address population needs. Local contextual factors (population characteristics and needs as well as prior service configurations and their perceived quality) that led to the original establishment of the individual services may also have been important.

If standalone services were not able to reduce service utilisation, it is relevant to consider how well they address other population needs. Results of a separate study of the AHPMCTs demonstrated that there was no effect on access and only a non-significant reduction in perceived unmet need for AH PMC care (forthcoming). In addition, other studies in the structured review have reported reduced patient satisfaction with telephone consultations when these replace in-person consultations [4,8,9]. While this did not occur with the standalone services, this effect may have limited the potential of telephone triage to replace face-to-face GP consultations.

AH service provision has moved on in both Australia (with introduction of second and third phase trials) and in the UK, as described in the Introduction, since the conduct of this study. NHS Direct is now much less a standalone service than previously and is much better integrated with AH general practices. Impacts on utilisation though may not have changed. In a recent observational study of the new integrated out-of-hours service where NHS Direct provided the entry point for all AH services, the four AH exemplars did not demonstrate a reduction in overall demand when compared with the 10 control GP cooperatives and there was an increase in ambulance usage [16].

## Conclusion

Bearing in mind limitations of all current methods recording client utilisation, including client recall as used in this study, it can be concluded that telephone triage and adviceservices had small effects that differed depending upon their form and function. Standalone call centres with nurses using nurse-administered proprietary health call centre software, which aimed at addressing population needs showed some increases in service use. By comparison, embedded systems using other than these software packages, which aimed more at managing demand, showed, if anything, a decrease in service use and change in mix. It is concluded that the effects of telephone triage on service utilisation and mix are influenced by the type of telephone triage offered, the goals of the agency providing the service, as well as other local factors.

Actual word count 3626 (without references, tables, figures & abstract)

## Competing interests

The author(s) declare that they have no competing interests.

## Authors' contributions

DD made a substantial contribution to conception and design, acquisition of data, analysis and interpretation of data and was involved in drafting the manuscript. SED made a substantial contribution to acquisition of data, analysis and interpretation of data and was involved in revising the manuscript critically for important intellectual content. MK made a substantial contribution to data analysis and was involved in revising the manuscript critically for important intellectual content. MM made a substantial contribution to conception and design and was involved in revising the manuscript critically for important intellectual content.
